# Study on Alginate–Chitosan Complex Formed with Different Polymers Ratio

**DOI:** 10.3390/polym8050167

**Published:** 2016-05-04

**Authors:** Dominika Kulig, Anna Zimoch-Korzycka, Andrzej Jarmoluk, Krzysztof Marycz

**Affiliations:** 1 Department of Animal Products Technology and Quality Management, Wroclaw University of Environmental and Life Sciences, Chelmonskiego Street 37, 51-630 Wroclaw, Poland; dom.kulig@gmail.com (D.K); andrzej.jarmoluk@up.wroc.pl (A.J.); 2 Electron Microscope Laboratory, Department of Animal Hygiene and Ichthyology, Wroclaw University of Environmental and Life Sciences, Kozuchowska Street 5B, 51-630 Wroclaw, Poland; krzysztof.marycz@up.wroc.pl

**Keywords:** chitosan, sodium alginate, polyelectrolyte complex, TGA, DMTA, MALDI-TOF

## Abstract

Biomaterials based on polyelectrolyte complexation are an innovative concept of coatings and packaging production to be applied in a wide range of food products. The aim of this study was to obtain and characterize a sodium alginate–chitosan complex material with variable degree of polyion interactions by complexation of oppositely charged polysaccharides. In order to characterize polyelectrolyte complexes, theromogravimetric analysis (TGA), dynamic mechanical thermal analysis (DMTA), nuclear magnetic resonance (NMR), Fourier transform infrared spectroscopy (FT-IR), matrix-assisted laser desorption/ionization technique with time of flight analyzer (MALDI-TOF), and scanning electron microscopy (SEM) were performed. TGA analysis showed that thermal decomposition temperature depends on the polymer ratio (*R*) and thermal resistance of samples was improved by increasing chitosan dosage. Accordingly to DMTA results, polyelectrolyte complexation led to obtain more flexible and resistant to mechanical deformation materials. Comparative analysis of the FTIR spectra of single polyelectrolytes, chitosan and alginate, and their mixtures indicated the formation of the polyelectrolyte complex without addition of reinforcing substances. MALDI-TOF analysis confirms the creation of polyelectrolyte aggregates (~197 Da) in samples with *R* ≥ 0.8; and their chemical stability and safety were proven by NMR analysis. The higher *R* the greater the number of polyanion–polycation aggregates seen in SEM as film morphology roughness.

## 1. Introduction

Polyelectrolyte complexes are growing in importance as materials for encapsulation of various types of substances, as a polymer matrix for controlled drug release systems, and as membranes in water treatment processes. The increased entropy associated with the release of counter-ions and the strong electrostatic attraction between oppositely charged polymers lead to the formation of polyelectrolyte complexes [1,2,3,4,5]. Materials made from chitosan (cationic polyelectrolyte) and sodium alginate (anionic polyelectrolyte) are examples of such complexes. Chitosan is a linear polysaccharide consisting of d-glucosamine and an *N*-acetyl glucosamine unit. It is produced by the deacetylation of chitin, a major component of the shells of crustaceans. This biopolymer contains three types of reactive functional groups: an amino/acetamide group, and both primary and secondary hydroxyl groups at the C-2, C-3 and C-6 positions. Chitosan has been of interest in the medical and food industries due to its numerous biological properties, including antimicrobial, antioxidant, metal chelating, lipid binding, hypocholesterolemic, immunostimulating, antitumor, anti-inflammatory, and anticancer effects [6]. Because of multidirectional biological activities, chitosan is used in complex composition instead of other cationic natural polymers (e.g., gelatine, dextran, and cellulose). Alginate is a polysaccharide newly used in industrial food coating to form a gel by binding divalent metal ions (Mg^2+^, Mn^2+^, Ca^2+^, Sr^2+^, Ba^2+^, Cu^2+^, and Pb^2+^). The ability to bind cations is strongly dependent on the binding affinity of the polysaccharide to the metal [7,8] and the chemical composition of an alginate [9]. The formation of an egg-box structure is enhanced with increasing content of α-guluronic acid in its structure. The ratio of d-mannuronic acid (M) and α-guluronic acid (G) in alginate had no influence on polyelectrolyte complexation [4]. It is possible to use alginates with a predominance of M blocks in the chain (e.g., derived from algae species: *Macrocystis pyrifera* and *Ascophyllum* sp.) in the polyelectrolyte complexation process. Furthermore, polymeric materials made from two or more substances may be used to exploit the specific functional properties of each individual component and to minimize their disadvantages.

The polyelectrolyte network is formed by the interaction between the dissociated functional groups: an anionic carboxyl group of alginate and a cationic amino group of chitosan, intra- and inter-chain hydrogen bonding between different parts of the polysaccharides’ structures and between already created aggregates of alginate–chitosan particles [10]. Complexation of polyelectrolytes leads to coacervate and hydrogel formation or precipitation. The occurrence of these processes depends on the reagent concentration, ionic strength, pH, temperature, order of mixing, flexibility of polymers, and chemical composition of polymers [1,4,5]. Polyelectrolyte complexes from chitosan and sodium alginate may be formed by various methods; a one-step process involves the gradual addition of chitosan solution to alginate [11]. An alternative method is to produce a multilayer coating (layer-by-layer, bilayer): chitosan solution is poured out/sprayed on to a dried alginate coating, or alginate is immersed in a chitosan solution and rinsed with distilled water after a certain time of interaction [12]. Properties of polyelectrolyte complex fibers [13], beads [14], sponges/scaffolds [15], nanolayered PET films [16], drug-loaded membranes [17], membranes for soft tissue engineering [18], and multilayers [19,20] have already been investigated. Introduction of sodium alginate–chitosan polyelectrolyte complexes into food coating and processing systems is an innovative idea.

The aim of this study was to explore the effect of sodium alginate enrichment in biologically active chitosan and form a material with novel properties by polyelectrolyte complexation. Analysis of the physico-chemical changes occurring as a result of polymers’ interaction and of the stability of the obtained complex during different kinds of processing is necessary because of potential food applications. To our knowledge, polyelectrolyte complexation using wide range of sodium alginate/chitosan ratios and characterization of their variable features is novel and has not been published yet. In addition, the conditions of e.g., TGA and DMTA analysis have been selected to meet the requirements of industrial food processing technology. Controlling of polyanion–polycation binding degree by modifying polymer ratios and understanding the structure and dynamic properties of this system will help to introduce it to the food market in the future. It also allows for a more accurate understanding of the polyelectrolyte complexation process. TGA, DMTA, NMR, FT-IR, MALDI-TOF, and SEM analyses were used to monitor the impact of specific factors on the formed polysaccharide network.

## 2. Experimental

### 2.1. Materials

Chitosan (molecular weight (*M*_w_) ≈ 50–190 kDa, degree of deacetylation (DD) = 82.84% (measured by titration method), viscosity of 0.5% soln. *w*/*v* in 1% soln. lactic acid: 0.03 ± 0.01 Pa∙s) was supplied by Sigma-Aldrich (St. Louis, MO, USA). Sodium alginate extracted from *Laminaria digitata* (viscosity of 0.5% soln. 0.04 ± 0.00 Pa∙s, particle size max. 5% > 400 μm, M:G ratio = 1.4) was supplied by Danisco (Copenhagen, Denmark). Glycerol was from Sigma-Aldrich and 80% lactic acid was purchased from Purac (Gorinchem, The Netherlands).

### 2.2. Preparation of Sodium Alginate–Chitosan Hydrosols

Sodium alginate powder (0.5%, *w*/*v*) was dissolved in deionized water by stirring (RW 20 digital, IKA®-Werke GmbH & Co. KG, Staufen, Germany) at 350 rpm for 24 h. Similarly, 0.5% of chitosan solution (*w*/*v*) was prepared in a 1% solution of lactic acid (*v*/*v*). Pure sodium alginate (ALG) solution (0.5% *w*/*v*) and pure chitosan (CH) solution (0.5% *w*/*v*) were reference samples and were used to produce a sodium–alginate polyelectrolyte complex (ACH). Polycation and polyanion solutions (*v*/*v*) were mixed in different volume ratios and homogenized (T18 basic, ULTRA TURRAX, IKA®-Werke GmbH & Co. KG, Staufen, Germany) for 1 min in the proportions described in Table 1. At the end of the homogenizing process, the glycerol was added as a plasticizer (30% of dry weight of used polysaccharide) to sodium alginate–chitosan blends and their references. Finally, the composition was degassed using sonification to form bubble-free solutions. The pH of the obtained ALG, CH, and ACH hydrosols was measured. Hydrosols were used as film-forming solutions and tested for MALDI-TOF analysis.

### 2.3. Hydrogel Film Formation

The complex ACH hydrosols and their references (ALG and CH) at an amount of 80 cm^3^ were poured out on leveled, degreased Teflon plates with dimensions of 8 cm × 20 cm. Plates were placed in a climatic chamber (KBF-LQC240 model, BINDER GmbH, Tuttlingen, Germany) for 48 h at 25 °C and relative humidity 60%, to evaporate excess of solvent. The dried films were peeled off from plates and cut into appropriate pieces for chosen analysis: TGA, DMTA, NMR, FT-IR, and SEM.

### 2.4. Thermal Gravimetric Analysis (TGA)

The thermal gravimetric analysis (TGA) was used to measure the thermal stability of complexed chitosan–sodium alginate films. Hi-Res TGA 2950 Thermogravimetric Analyzer from TA Instruments Company (Tokyo, Japan) was used. The analyses were made by increasing the temperature from room temperature to 300 °C in an inert nitrogen atmosphere with a flow rate of 60 mL/min and warming rate of 10 °C/min.

### 2.5. Dynamical Mechanical Thermo Analysis (DMTA)

Dynamical mechanical measurements were carried out with a DMTA Mk III instrument (Rheometric Scientific, Loughborough, UK), at a frequency of 1 Hz and a heating rate of 2 °C/min, from −80 to +50 °C. The values of dynamic storage modulus, loss modulus, and phase angle were measured.

### 2.6. Nuclear Magnetic Resonance (NMR)

Nuclear magnetic resonance measurements of solid state with cross-polarization spinning under magic-angle technique CP MAS NMR were performed on an Avance III 400 MHz spectrometer (Bruker Corporation, Billerica, MA, USA). The spectra for the ^13^C nuclei were taken with 100.61 MHz frequency and for ^1^H nuclei with 400.15 MHz frequency in the broadband probe MAS BB DVT which enabled of use zirconia rotors (ZrO_2_) with 4 mm diameter. Isotopically labeled l-tyrosine (Tyr) was used to select the parameters for ^13^C CP MAS NMR spectra including optimizing for Hartmanna–Hahna conditions. The most important spectroscopic parameters: ^13^C CP MAS spectra: measurement temperature 298 K, rotation speed 8 kHz, relaxation time 3 s, pulse 90° for ^1^H 4 μs, contact time 2 ms, spectral width SWH = 40 kHz, TD = 3.5 k, SPINAL decoupling type; ^1^H MAS spectra: measurement temperature 298 K, rotation speed 8 kHz, relaxation time 2 s, pulse 90° for ^1^H 4 μs, spectral width SWH = 40 kHz, TD = 16 k.

### 2.7. Fourier Transform Infrared Spectroscopy (FT-IR)

The FTIR spectra of pure and complex membrane were registered in Infinity AR60 spectrometer (ATI Mattson, Matson, WI, USA). The spectra were recorded between 450 and 4000 cm^−1^ by 64 scans at a resolution of 2 cm^−1^.

### 2.8. Matrix-Assisted Laser Desorption/Ionization Technique with Time of Flight Analyzer (Maldi-Tof)

Samples were prepared by mixing 10 μL of the test hydrosols with 10 μL of matrix solution (aqueous solution of 2.5-dihydroxybenzoic acid at 10 mg/mL concentration). Subsequently 2 μL of the prepared solution was applied on a plate to speed solvent evaporation. In the next step, the plate was introduced into the mass spectrometer ion source Voyager-Elite from PerSeptiveBiosystems Company (Framingham, CT, USA). As the ionization technique, desorption/ionization matrix-assisted laser (matrix-assisted laser desorption/ionization—MALDI) was used. The wavelength of the laser radiation—337 nm, the energy of the laser beam—was slightly above the level needed to get the signals; the accelerating voltage (20 kV) was set. Positive ions were subjected to registration using time of flight of ions analyzer (time of flight—TOF) with ion reflection; the recorded mass range (*m*/*z*) was between 300 and 3500. However, in the mass range 300 to 500 *m*/*z* intense peaks dominate the matrix, therefore mass range from 500 *m*/*z* was taken into account. Mass spectrum is the sum of 200 spectra, each consisting of two laser shots of randomly selected sampling points (200 spectra × 2 shots = 400 laser shots). External mass calibration was used, made on the basis of a defined reference mixture of polyethylene glycols spectrum.

### 2.9. Scanning Electron Microscopy

Morphologies of the prepared materials were characterized using an EVO LS15 (Zeiss, Oberkohen, Germany) with an accelerating voltage of 20 kV. All specimens were frozen in liquid nitrogen and broken into pieces to expose the cross-section region of coatings. Then samples were dehydrated in graded ethanol solutions before applying critical point drying. The samples were dried using an E3100 Critical Point Dryer (Quorum Technologies, Ltd., West Sussex, UK). The SEM observations were performed after spattering the samples with gold for short bursts totaling 140 s, to avoid coating heating, using a sputter coater Scancoat 6 (Edwards, London, UK).

### 2.10. Statistical Analysis

The interactional effects of two categorical variables, such as dose of sodium alginate and chitosan, were evaluated. Statistical analysis was performed with multivariate analysis of variance (ANOVA) using Statistica 10 (StatSoft, Krakow, Poland). Differences between mean values were identified by a Duncan test with a confidence level of *p* < 0.05. All experiments were performed in triplicate unless mentioned otherwise.

## 3. Results and Discussion

### 3.1. pH Measurement

The charge density, type of solvent, ionic strength, temperature, and pH affect complex formation and stability [4]. The pH of pure sodium alginate and chitosan solutions was 6.40 and 3.84, respectively. The dissociation constant of chitosan is 6.5–6.6 and the pKa-values of mannuronic and guluronic acid monomers of alginate were found to be 3.38 and 3.65, respectively [21]. A polyelectrolyte complex reaction can occur between the dissociated functional groups, the anionic carboxyl group of alginate and cationic amino group of chitosan. The pH values have to be between the pKa of chitosan and the pKa of alginate monomers to form a complex, which was achieved in the present study. The average pH of sodium alginate–chitosan polyelectrolyte complex hydrogels was 3.92 ± 0.07. The applied reaction conditions did not provide separation of the individual phases or precipitation.

### 3.2. Thermogravimetric Analysis (TGA)

**Figure 1:**
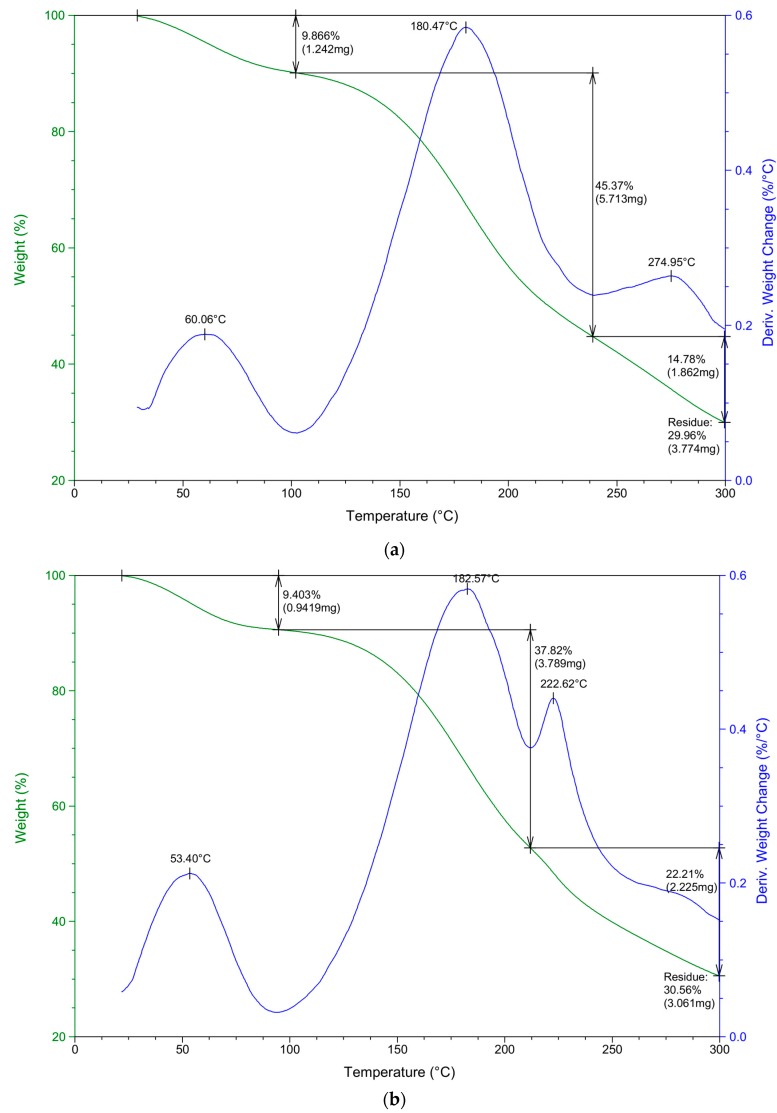
Thermal decomposition curves of sodium alginate–chitosan complexed samples (**a**) ACH-1; (**b**) ACH-9.

Thermal decomposition process of complexed materials and their pure counterparts was assessed by thermogravimetric analysis (TGA). The thermal decomposition curves were provided as the inflection points depicting the peak maximum to evaluate the degradation characteristics (Table 2). Simultaneously, percentage mass loss during thermal decomposition was investigated (Table 3). Furthermore, exemplary thermal decomposition curves of sodium alginate–chitosan complexed samples (ACH-1 and ACH-9) are presented in Figure 1. The obtained results showed that a pure sodium alginate sample undergoes a two-stage thermal degradation process, while chitosan and polyion blends are degraded in a three-stage process (Table 2). The first stage of the decomposition process occurred between 53 and 71 °C, which is due to the loss of mass through vaporization of volatile components, such as free water present in the coating. It was observed that samples with the highest amount of chitosan (ACH-1, ACH-2, and ACH-4) needed a higher temperature to release absorbed water than samples with a lower concentration of this polysaccharide (*p* < 0.05). This is a result of stronger immobilization of water molecules between chitosan chains. All sodium alginate–chitosan films showed lower free water mass lost at the first step of decomposition than uncomplexed chitosan and sodium alginate samples. This may be related to the lower concentration of free water in the complexed material. Considering the structure of chitosan and alginate, it can be seen that water molecules can be bound by three polar groups: amine, carboxyl, and hydroxyl are in chitosan, sodium alginate, and both polymers’ structure, respectively [22]. When a reaction between the amine groups of chitosan and the carboxylic groups of sodium alginate occurs, the interactions of polar groups with those functional groups of polysaccharides are impeded and fewer groups of polysaccharides can react with water molecules, resulting in lower water absorption capacity. The second peak (responsible for major weight loss) showed release of water bounded to the functional groups of both polymers, which was not completely removed in the first step of dehydration. Also, degradation of carboxylic groups present in the sodium alginate structure appeared at the temperature range 178–190 °C. The thermal stability of materials is determined by major mass loss, after which thermal degradation starts. The thermal stability of sodium alginate increased due to complexation with chitosan. The greatest changes were observed after exceeding 200 °C. The last inflection point of the temperature range 270–280 °C was characteristic for deacetylation and partial depolymerization of the chitosan chain and was observed in a pure chitosan sample and the complexed sodium alginate–chitosan material with the highest amounts of mentioned polysaccharides (ACH-1, ACH-2, and ACH-4). An excess of chitosan in these samples’ composition resulted in the formation of a stabilizing/protective layer around aggregated ALG-CH polyelectrolyte structures, which contributed to improvements in their thermal resistance. A third inflection point was not observed in both pure polyelectrolytes samples (sodium alginate and chitosan), but it occurred in some complexed samples (ACH-3, ACH-5, ACH-6, ACH-7, ACH-8, and ACH-9), which may be associated with the decomposition of the polyelectrolyte complex and may be considered proof of its presence in modified materials. The final residue at the maximum analysis temperature (300 °C) for all complexed samples was about 30% of their total weight (Table 3).

### 3.3. Dynamic Mechanical Thermal Analysis (DMTA)

**Figure 2:**
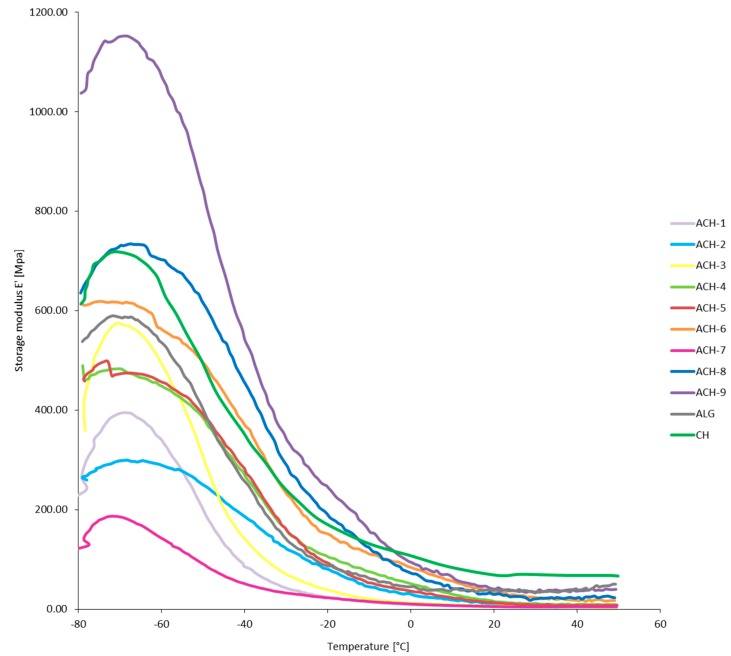
Storage modulus (*E*’) as a function of temperature of complexed materials (ACH-1–ACH-9) and pure sodium alginate (ALG) and chitosan (CH) films.

**Figure 3:**
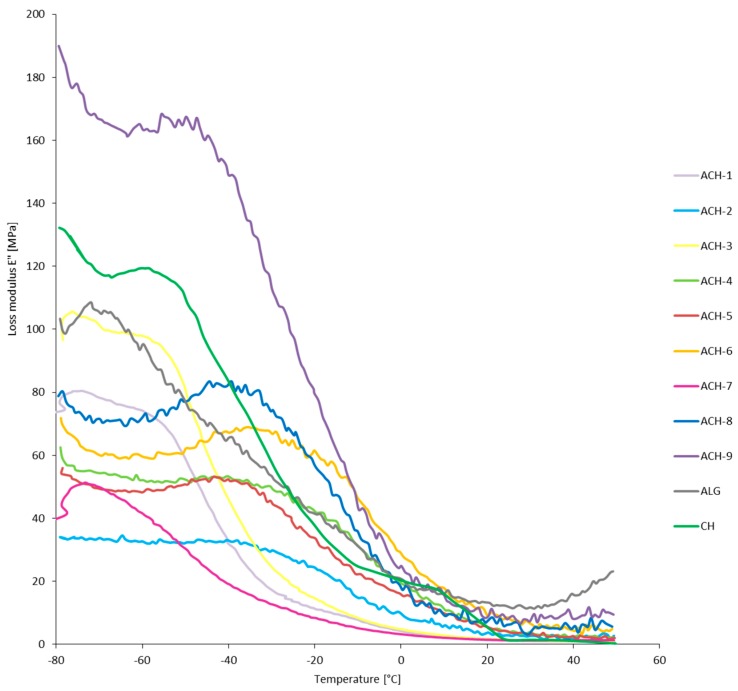
Loss modulus (*E*”) as a function of temperature of complexed materials (ACH-1–ACH-9) and pure sodium alginate (ALG) and chitosan (CH) films.

**Figure 4:**
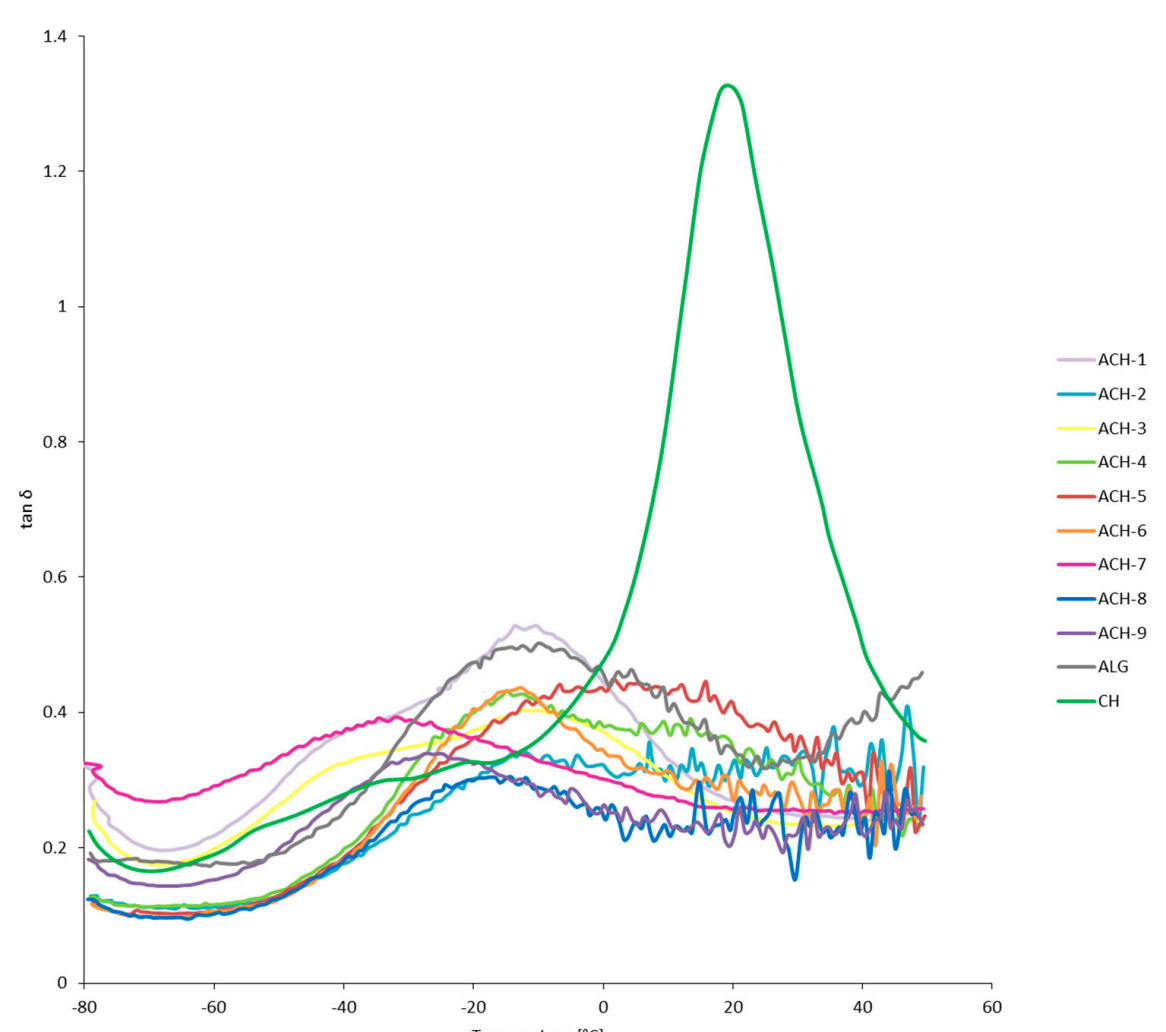
Tan δ as a function of temperature of complexed materials (ACH-1–ACH-9) and pure sodium alginate (ALG) and chitosan (CH) films.

The mechanical and viscoelastic properties of materials in temperature function were evaluated by DMTA analysis. The direction of changes in storage modulus, loss modulus, and loss tangent of ALG, CH, and ACH samples are presented in Figure 2, Figure 3 and Figure 4, respectively. The first component of the analysis is the storage modulus (*E*’), determining the elastic response of a material to a given load. Mixing oppositely charged polyelectrolytes resulted in a significant increase of the storage modulus in ACH-8 and ACH-9 films. Pure polysaccharides showed reduced *E*’ values compared to the samples with the highest polymer ratio (*R* ≥ 0.8). Formation of additional bonds between the chains of the polysaccharides makes material stiffer and more resistant to mechanical deformation, especially in low-temperature conditions. However, samples ACH-1–ACH-7 (*R* ≤ 0.6) exhibited decreased values of storage modulus compared to ALG and CH samples, confirming the effect of the polymers ratio on the mechanical and viscoelastic properties of the coating. Loss modulus (*E*”) is the ratio of the amplitude of viscous tension to the strain amplitude. It indicates how much energy has been distributed in the form of heat during deformation. It was observed that the fall in E” shifted to higher temperature after polyions’ interaction and continued with decreasing polymer ratio of polyelectrolytes. An exception was sample ACH-7, characterized by the lowest values of the storage and loss modulus, which means it most easily undergoes mechanical deformation. Sample ACH-9, with the highest polymer ratio (*R* = 1.0), was characterized by the greatest loss modulus. The size of the loss tangent gives information about the number of elastic and viscous constituents represented in the sample, described by the equation tan δ = *E*”/*E*’, where δ is the phase angle. The phase angle is 90° when the material is fully viscous and is 0° for a completely flexible material. Viscoelastic material is when 0° < δ < 90°. The loss tangent, δ, is an index of the solid-like (≤1) or liquid-like (≥1) behavior of the material [23]. All polyelectrolyte ACH samples and the ALG reference were characterized by loss tangent <1°, which confirms their solid-like behavior. It was observed that polyelectrolyte complex samples and the alginate reference had retained their plastic properties after refreezing. During the analysis, as a result of the transition from negative to positive temperatures, liquefaction of the chitosan sample occurred, which corroborated the high tan value (tan δ > 1°). Significant diversity in DMTA results was noted for the samples tested in negative and positive temperatures. As a result of freezing, all samples (ACH-1–ACH-9, ALG, and CH) analyzed in low-temperature conditions showed a stiffer structure, which can be observed in the high *E*’ and *E*” values and the large difference between the values of both modules. At positive temperatures the film structure is flexible and gel-like, which was confirmed by similar *E*’ and *E*” values.

### 3.4. Nuclear Magnetic Resonance (NMR)

**Figure 5:**
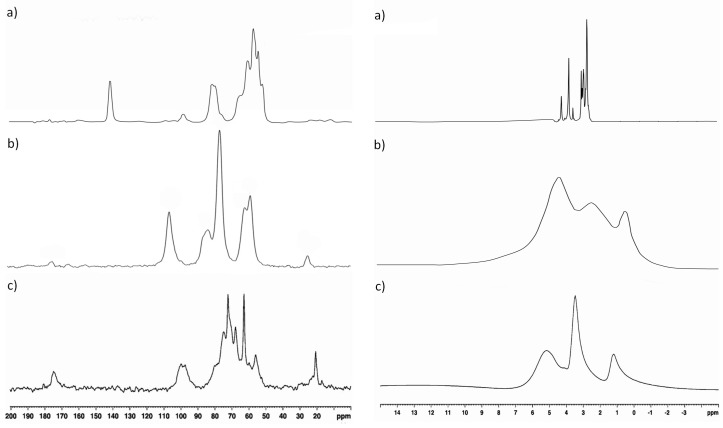
^13^C CP MAS NMR (**left** column) and ^1^H MAS NMR (**right** column) spectrum of (**a**) sodium alginate film; (**b**) chitosan film; and (**c**) sodium alginate–chitosan polyelectrolyte complex film.

^13^C CP MAS NMR (left column) and ^1^H MAS NMR (right column) spectra of sodium alginate, chitosan, and a sodium alginate polyelectrolyte complex film are shown in Figure 5a–c, respectively. Lack of differentiation in the aliphatic range for all ^13^C CP MAS NMR spectra of complexed samples ACH-1–ACH-9 was observed; therefore, sample ACH-9 was presented as representative of all complexed samples. According to Salomonsen *et al.* [24], carbon spectra consist of three regions of carbon signals: pyranose (60–90 ppm), anomeric (90–110 ppm), and carboxyl (172–180 ppm). A small peak near 176 ppm was observed, which is due to the presence of carbonyl carbon of –COCH_3_ from chitosan structure and alginate salt. A peak centered at 100 ppm was characteristic for the C1 carbon atom of the guluronate and mannuronate unit and the C1 carbon assigned to the acetylated amino group from the chitosan structure. Residual lactic acid was assigned to small signals at 20 ppm and 180 ppm. Peak intensities in the region 50–90 ppm are assigned to C2–C6 glucopyranose carbons from chitosan, which overlap with signals corresponding to carbons of guluronic and mannuronic acid of sodium alginate. Similarly to ^13^C CP MAS NMR, no significant changes in the ^1^H MAS NMR spectrum were found between samples with different polymer ratios, which indicates a certain stability and homogeneity of obtained materials. A signal centered at 1.2 ppm is assigned to the CH_3_ group belonging to a residual of lactic acid and was observed in both chitosan and polyelectrolyte ACH blend spectra. A chemical shift of the hydrogen of methyl moieties (from acetylated amino group of chitosan) was observed near 2.0 ppm. The carboxyl group of sodium alginate reacted ionically with the chitosan amino group during complexation, which resulted in the disappearance of the H–Ac peak in all blended ACH samples. According to the study of Jančiauskaitė *et al.* [25], the intense signal observed between 3.5 and 4.0 ppm is assigned to alginate H2–H5 moieties and H2–H6 protons of the chitosan chain. The broad signal observed in the ^1^H MAS NMR spectrum between 4 and 7 ppm is assigned to solvent protons in the form of water and glycerol, which was used as a plasticizer. These signals overlap with the signals designated the H1 proton of guluronic acid, characteristic of the sodium alginate chain, and hydrogen bonded to the anomeric carbon (C1) from the glucosamine unit. Glycerol was added as a constant factor to obtain better mechanical properties of experimental films and to inhibit the precipitation process (by increasing the distance between polymer molecules and reducing the attraction between polyions), which occurs often as a result of rapid reaction between polyions [26].

### 3.5. FT-IR Analysis

**Figure 6:**
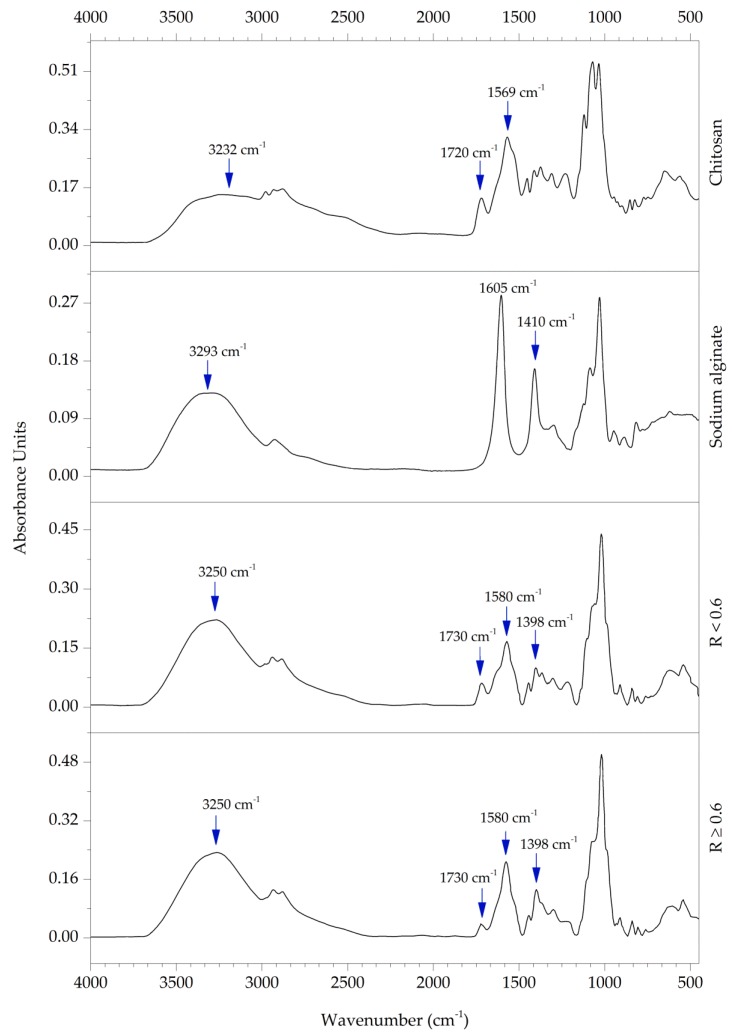
The FT-IR spectra of chitosan, sodium alginate, and their blends with polymer ratios *R* < 0.6 and *R* ≥ 0.6.

FT-IR spectra of chitosan (CH), sodium alginate (ALG), and their polyelectrolyte blend (ACH) are shown in Figure 6. The spectrum of CH presents a broad and intense band centered at about 3232 cm^−1^ and corresponds to stretching vibrations from overlapping of the O–H and N–H bonds. Peaks observed at 2973, 2933, 2881, 1413, 1310, and 1230 cm^−1^ were due to symmetric or asymmetric CH_2_ stretching vibrations of pyranose ring. Residual lactic acid is noticeable at 1720 cm^−1^, corresponding to carbonyl vibration of the carboxylic acid, which was confirmed by Lawrie *et al.* [12]. Characteristic absorption bands of chitosan are usually seen between 1649 and 1652 cm^−1^ and 1558–1598, corresponding to C–O stretching (amide I) and N–H bending (amide II), respectively [10,27,28]. Those bands shift and overlap each other, creating s strong peak at 1569 cm^−1^, which could be related to the lactic acid presence in the ACH blend. Absorption bands at 1121 cm^−1^ (antisymmetric stretching of the C–O–C bridge), 1071 cm^−1^, and 1035 cm^−1^ (skeletal vibrations involving the C–O stretching) are characteristic of the polysaccharide structure [10,28]. The absorption bands of alginate near 1605 and 1410 cm^−1^ are associated with the asymmetric and symmetric stretching vibrations of carboxylate anions, respectively, as others observed [29,30]. The sodium alginate spectrum also shows a strong and broad band at 3293 cm^−1^ related to O–H stretching and weak aliphatic C–H stretching band at 2926 cm^−1^. Due to its polysaccharide structure bands around 1298 cm^−1^ (C–O stretching), 1124 cm^−1^ (C–C stretching), 1086 cm^−1^ (C–O stretching), 1031 cm^−1^ (C–O–C stretching), and 948 cm^−1^ (C–O stretching) were noted [10]. In FTIR spectra of mixed oppositely charged polysaccharides, we observed changes of some bands’ placement and disappearance or appearance of new peaks in comparison to single alginate or chitosan. The complexed material has a narrower, more intense band at about 3250 cm^−1^, which is caused by forming new hydrogen bonds between –OH and –NH_2_ groups in chitosan and –C=O and –OH groups of sodium alginate [14]. Bands assigned to motions of carboxylate salt group were invisible after complexation. This disappearance is caused by the lower content of alginate and chitosan excess in all ACH composites. New peaks around 1580 cm^−1^ and 1730 cm^−1^ were observed in all blends. A peak at 1580 cm^−1^ has greater intensity in samples with *R* ≥ 0.6. A peak at 1730 cm^−1^ possibly corresponded to asymmetric stretching of –COO groups, which confirms polyelectrolyte complex formation. However, when the polymer ratio was ≥0.6 the peak intensity was observed at 1398 cm^−1^, confirming stronger electrostatic interaction in samples with a polymer ratio closer to the stoichiometric balance (*R* ≈ 1.0). The presence of the aforementioned bands in the ACH polymer mixture indicates the appearance of ionic bonds between chitosan amine groups and carboxyl group of alginate.

### 3.6. Matrix-Assisted Laser Desorption/Ionization Technique with Time of Flight Analyzer (MALDI-TOF)

**Figure 7:**
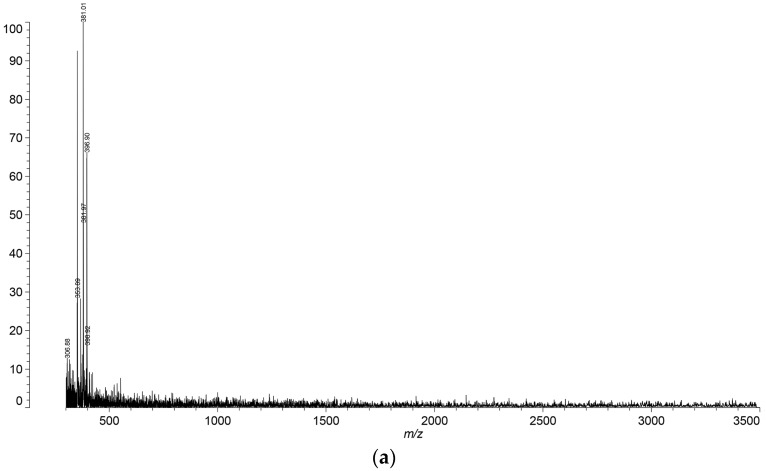

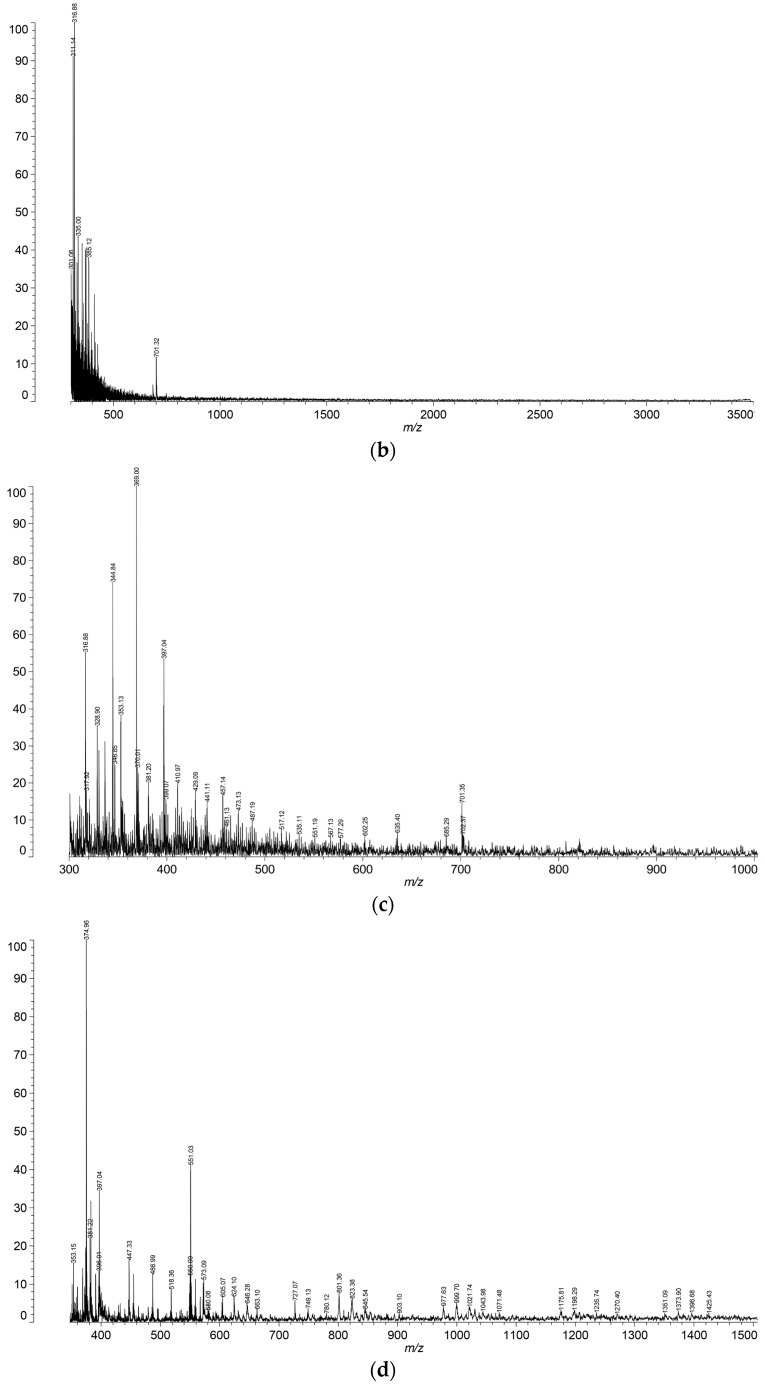
(**a**) MALDI-TOF spectra of sodium alginate sample (ALG); (**b**) MALDI-TOF spectra of chitosan sample (CH); (**c**) MALDI-TOF spectra of sodium alginate–chitosan complexed sample (ACH-6); (**d**) MALDI-TOF spectra of sodium alginate–chitosan complexed sample (ACH-8).

Exemplary MALDI-TOF spectra of sodium alginate (ALG), chitosan (CH), and complexed samples ACH-6 and ACH-8 are shown in Figure 7a–d, respectively. The signals observed in the chitosan spectrum at 685 and 701 *m*/*z* are due to the presence of sodium and potassium *N*-glucosamine oligomers, respectively. Those signals were not observed in the sodium alginate spectrum. Groups of signals of ACH-8 (Figure 7d) and ACH-9 variants were seen in the range of 600–1600 *m*/*z* and exhibited a constant difference in *m*/*z* to each other (Δ *m*/*z* = 197 Da). Moreover, in each group, signals differed by 22 Da, which may be associated with the presence of sodium ions of particular structures. Those groups of signals were probably attributed to the formation of sodium alginate–chitosan complex aggregates by creating additional ionic bonds between already created polyelectrolyte complex particles. The aforementioned groups of signals were not observed in samples ACH-1–ACH-7, characterized by lower polymer ratios (Figure 7c). According to Figure 6d, aggregation behavior near the stoichiometric point of the sodium alginate–chitosan complex (*R* ≥ 0.8) was observed. Sæther *et al.* [4] noticed that in an excess of the one of the major components, small and non-aggregating complex particles form due to the formation of a stabilizing shell around polyanion–polycation particles. The authors explained that local patches with a net charge not equal to zero can participate in electrostatic-mediated interactions with oppositely charged patches on other sodium alginate–chitosan clusters [4]. Similar findings were published for chitosan and dextran sulfate by Drogoz *et al.* 2007 [31] and Schatz *et al.* 2004 [32]. However, these studies focused on light scattering techniques, while confirmation of this dependence through MALDI-TOF analysis is a novel proposal.

### 3.7. Scanning Electron Microstructure (SEM)

**Figure 8:**
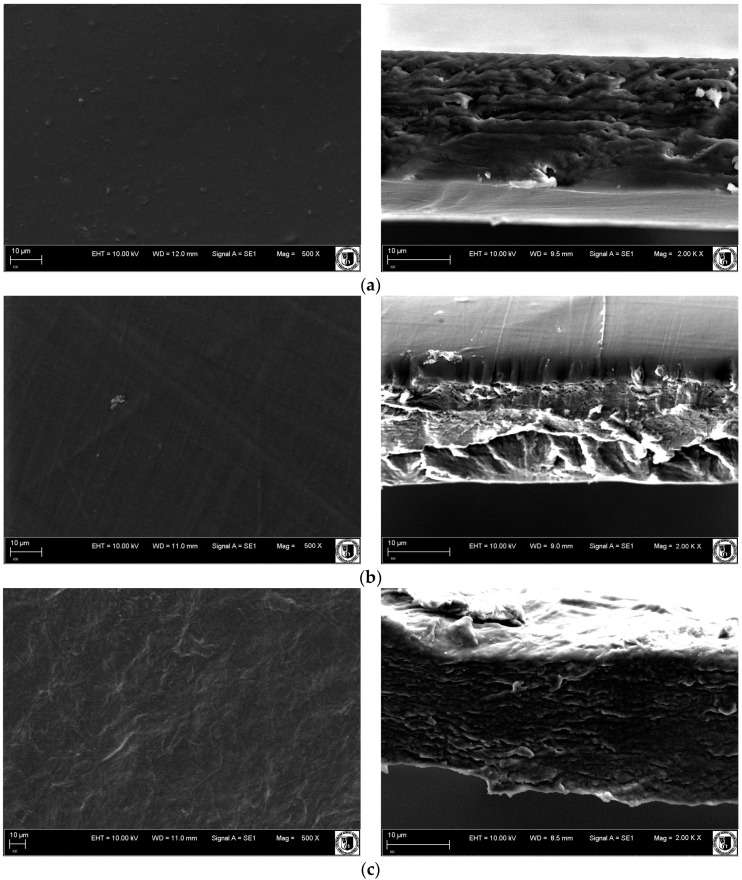

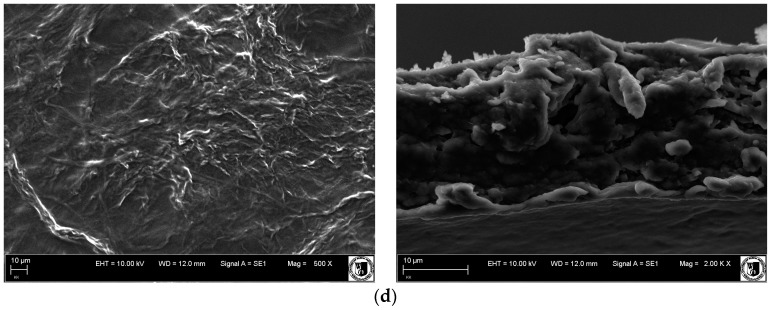
SEM images of surface (**left** column) and cross-section (**right** column) morphology of (**a**) sodium alginate; (**b**) chitosan; and complex films produced with various polymers ratios: (**c**) ACH-4 and (**d**) ACH-9. Magnification: surface 500 X/ cross-section 200 kX.

The surface and cross-section morphology of the ALG, CH, and ACH films are shown in Figure 8. The morphology of blended ACH films was less homogenous than ALG and CH. Similar descriptions of the microstructure of sodium alginate–chitosan based films examined with scanning electron microscopy have been reported by other authors [29,33]. Polyelectrolyte complex films showed irregular, fibrous structures of surface and rough cross-section morphology, with pores and clusters of sodium alginate–chitosan aggregated particles. It was observed that complex aggregates appear in micrographs as segments with round/spherical structures. It was found that with increasing polymer ratio more polyanion–polycation complex aggregates were formed, which can be seen as an increase in the structure’s irregularity. The differences in the appearance of the ACH films’ surface and cross-section are also due to more rapid ionic interaction in the stoichiometric region (*R* ≈ 1.0). A more uniform and compact cross-section structure was observed in samples containing an excess of unreacted chitosan, which can be caused by its uniform morphology.

## 4. Conclusions

In the presented research viscous and macroscopically homogeneous sodium alginate–chitosan hydrogel structures were obtained. The results of the analyses provide very detailed information about characteristics and changes caused by polyelectrolyte complexation of natural polymers. The desirability of connecting alginate and chitosan due to the improvement of their thermal, chemical, and mechanical properties and stability was confirmed. Properties of the chosen polysaccharides complement each other and, as a result, their functionality has been upgraded. Sodium alginate’s addition to chitosan improves its flexibility. Simultaneously, chitosan’s presence in a sample provides thermal resistance. Interactional acting of combined polymers positively affects the mechanical resistance of the sample. A stable polymeric structure of alginate–chitosan was obtained due to ion interaction. Applied reaction conditions allow for polyelectrolyte complex formation, which was confirmed by physico-chemical analysis of films (FT-IR, TGA, NMR, and MALDI-TOF analysis of hydrosols). It was proven that ALG-CH complex features are strongly dependent on the polymer ratio/binding degree and can be easily controlled and adjusted to the purposes of the application. Polyelectrolyte complexation precludes the need for additional chemical cross-linkers (e.g., glutharaldehyde). Complexation is not toxic, cheap, or complicated to achieve in industrial conditions. The obtained sodium alginate–chitosan complexes could be interesting for implementation in the food co-extrusion process. In this process core material (meat dough, textured fruit or vegetable pulp) is simultaneously extruded with a continuous flow of a thin surface layer of polymeric material. The easily controlled binding degree could help to adjust hydrosol/film properties to the different types and features of coated food products and their further processing.

## Figures and Tables

**Table 1:** Experimental design showing samples’ compositions and coding.

Variants	Volume Proportions of Hydrosols	Polymers Ratio
	ALG/CH	*R*
ACH-1	3/15	0.20
ACH-2	4/15	0.27
ACH-3	3/10	0.30
ACH-4	5/15	0.33
ACH-5	4/10	0.40
ACH-6	5/10	0.50
ACH-7	3/5	0.60
ACH-8	4/5	0.80
ACH-9	5/5	1.00
ALG	1/0	-
CH	0/1	-

**Table 2:** Thermal decomposition temperatures (based on inflection points) of pure polyelectrolytes: sodium alginate (ALG), chitosan (CH) and sodium alginate–chitosan polyelectrolyte complexes (ACH-1–ACH-9).

Sample	Inflection Point 1 (°C)	Inflection Point 2 (°C)	Inflection Point 3 (°C)	Inflection Point 4 (°C)
ALG	64.8 ± 0.4 ^e^	162.5 ± 0.8 ^c^	N	N
CH	71.7 ± 0.3 ^g^	181.3 ± 0.7 ^a^	N	279.2 ± 0.5 ^d^
ACH-1	60.1 ± 0.2 ^e^	180.9 ± 0.4 ^a^	N	275.6 ± 0.6 ^c^
ACH-2	58.1 ± 0.3 ^c^	182.5 ± 0.5 ^b^	N	270.5 ± 0.2 ^a^
ACH-3	56.7 ± 0.3 ^a^	180.9 ± 0.7 ^a^	211.8 ± 0.6 ^c^	N
ACH-4	58.4 ± 0.8 ^c^	191.2 ± 0.8 ^f^	N	273.5 ± 0.6 ^b^
ACH-5	56.9 ± 0.5 ^a^	183.7 ± 0.7 ^e^	208.7 ± 0.2 ^b^	N
ACH-6	54.0 ± 0.5 ^b^	182.6 ± 0.4 ^b^	222.2 ± 0.3 ^a^	N
ACH-7	55.0 ± 0.4 ^d^	181.2 ± 0.6 ^a^	222.4 ± 0.5 ^a^	N
ACH-8	56.3 ± 0.4 ^a^	178.5 ± 0.3 ^d^	222.5 ± 0.4 ^a^	N
ACH-9	54.2 ± 0.8 ^b^	182.6 ± 0.3 ^b^	222.1 ± 0.5 ^a^	N

N—no peak observed, values with different letters (^a–g^) within the same column differ significantly (*p* < 0.05).

**Table 3:** Percentage mass loss and residue of pure polyelectrolytes: sodium alginate (ALG), chitosan (CH) and sodium alginate–chitosan polyelectrolyte complexes (ACH-1–ACH-9) during thermal degradation.

Sample	Mass Loss 1 (%)	Mass Loss 2 (%)	Mass Loss 3 (%)	Mass Loss 4 (%)	Final Residue (%)
ALG	13.6 ± 0.7 ^g^	46.91 ± 0.5 ^c^	N	N	40.4 ± 0.2 ^d^
CH	13.6 ± 0.5 ^g^	23.19 ± 0.5 ^d^	N	24.2 ± 0.7 ^c^	40.3 ± 0.4 ^d^
ACH-1	10.1 ± 0.3 ^e^	45.12 ± 0.2 ^a^	N	14.3 ± 0.5 ^a^	30.2 ± 0.2 ^a,b^
ACH-2	9.3 ± 0.3 ^b–d^	46.85 ± 0.3 ^c^	N	14.0 ± 0.7 ^a^	30.5 ± 0.2 ^a,b^
ACH-3	9.6 ± 0.4 ^c–e^	44.92 ± 0.2 ^a^	15.3 ± 0.5 ^b^	N	30.7 ± 0.1 ^a^
ACH-4	8.6 ± 0.5 ^a,b^	44.5 ± 0.5 ^a^	N	15.2 ± 0.9 ^b^	30.9 ± 0.7 ^a^
ACH-5	11.2 ± 0.4 ^f^	31.8 ± 0.5 ^b^	17.5 ± 0.5 ^d^	N	29.4 ± 0.4 ^c^
ACH-6	8.9 ± 0.3 ^a–c^	38.7 ± 0.3 ^f^	22.1 ± 0.4 ^a^	N	29.9 ± 0.4 ^b,c^
ACH-7	11.1 ± 0.4 ^f^	31.3 ± 0.6 ^b^	28.9 ± 0.4 ^e^	N	27.9 ± 0.6 ^e^
ACH-8	8.4 ± 0.3 ^a^	40.0 ± 0.4 ^g^	20.10 ± 0.3 ^c^	N	30.9 ± 0.4 ^a^
ACH-9	9.7 ± 0.3 ^d,e^	37.8 ± 0.3 ^e^	22.28 ± 0.3 ^a^	N	29.9 ± 0.6 ^b,c^

N—no peak observed, values with different letters (^a–g^) within the same column differ significantly (*p* < 0.05).
